# Light-based electron aberration corrector

**DOI:** 10.1038/s41566-025-01760-8

**Published:** 2025-09-23

**Authors:** Marius Constantin Chirita Mihaila, Petr Koutenský, Kamila Moriová, Martin Kozák

**Affiliations:** Department of Chemical Physics and Optics, Faculty of Mathematics and Physics, Charles University, Prague, Czech Republic

**Keywords:** Adaptive optics, Quantum optics

## Abstract

Achieving atomic resolution in electron microscopy has historically been hindered by spherical aberration, a fundamental limitation of conventional electron lenses. Its correction typically requires complex assemblies of electromagnetic multipoles. Here we demonstrate that third-order spherical aberration in a cylindrically symmetric electron lens with an associated aberration coefficient of *C*_s_ ≈ 2.5 m can be compensated to near-zero via interaction with a shaped light field. By analysing distortions in the high-magnification point-projection electron images of optical standing waves, we quantify the spherical aberration before and after light-induced correction. The spatial distribution of the correction optical field is precisely characterized in situ using ultrafast four-dimensional scanning transmission electron microscopy utilizing the transverse deflection of electrons induced by the optical ponderomotive force. Such a combined characterization and correction approach introduces a new paradigm for optical control in electron beams and opens a pathway towards compact and tunable light-based correctors for high-resolution electron microscopy.

## Abstract (web summary)

Irradiation with a pulsed Laguerre–Gaussian laser beam of charge one enables correcting the third-order spherical aberration of an electron beam.

## Main

Spherical aberration in an optical system causes rays that pass farther from the optical axis to be focused closer to the lens than those passing near the axis, resulting in a blurred focal spot and limited resolution^1^. In contrast to light optics, where spherical aberration results from the curved shape of lens surfaces and chromatic aberration stems from wavelength-dependent variations in the refractive index (both of which can be mitigated through tailored lens shapes or multi-element designs), the situation in electron optics is fundamentally different. Scherzer’s theorem predicts that electrostatic and magnetostatic fields in rotationally symmetric electron lenses cannot be configured to eliminate spherical aberration^1^.

For half a century, efforts to correct these aberrations have met limited success. Early multipole corrector designs demonstrated the possibility of compensation, but did not translate into actual improvements in resolution due to mechanical instability, electromagnetic interference and imperfect alignment^2^. The first successful implementation of spherical aberration correctors made in 1990^3^ revolutionized electron microscopy by enabling sub-ångström spatial resolution. Since then, the direct imaging of materials with atomic resolution has advanced materials science, nanotechnology and structural biology, and has improved precision in electron-beam lithography for the semiconductor industry^4^. However, the performance of electron aberration correctors is limited to the compensation of low-order aberrations because they do not offer arbitrary phase shaping capabilities.

In light optics, the advent of programmable and adaptive devices, particularly spatial light modulators (SLMs), has enabled the precise and dynamic control of optical wavefronts^5^. In electron optics, however, phase shaping technologies have emerged only recently^6–9^, operating through an analogy to refractive-index modulation in light optics. Here electrons must propagate through a solid-state structure, leading to unavoidable scattering losses and degradation of the beam quality. To solve these limitations, one can utilize the interaction between electrons and photons to modulate the phase of electron beams either in the vicinity of a nanostructure^10–26^ or in free space^27–36^. Free-space modulation of electron wavefronts based on ponderomotive interaction offers distinct advantages, inherently minimizing decoherence and avoiding electron loss. Moreover, combining the light shaping capabilities of SLMs with the electron phase modulation has recently enabled the adaptive phase control of electrons^37,38^.

The underlying mechanism of the free-space electron–light interaction applied in this work can be understood either semi-classically, as a phase modulation of an electron wave by the electromagnetic field of light, or quantum mechanically, as stimulated Compton scattering^37,38^. In the former picture, the electron wave propagating through classical electromagnetic fields acquires a phase modulation proportional to the local light intensity. In the latter picture, the electron coherently acquires a position-dependent phase through the simultaneous absorption and emission of photons^37^. One of its most compelling applications is the dynamic correction of aberrations in electron lenses, a topic that has attracted important recent theoretical interest^37,39–45^.

Although proof-of-concept experiments^38^ demonstrated the transfer of spatial modulation from light to electrons, its application to electron aberration correction requires two major issues to be resolved.

The first critical requirement for aberration correction demonstration is a precise method that enables one to determine the coefficient of spherical aberration *C*_s_ during the corrector operation. Aberrations in electron lenses can be characterized using diffractogram analysis^46^ and Ronchigrams^47^, and when these are not applicable, shadow imaging with fine gratings serves as a valuable alternative^41,48^. In our study, we use a novel method, the point-projection imaging of an optical standing wave, which acts as an etalon sample composed of light. The distortion of the resulting image is a direct measure of *C*_s_. At the same time, the interaction of electrons with the optical standing wave occurs in a vacuum, preventing additional scattering or electron loss. However, the presence of other types of aberration may complicate the analysis.

The second challenge is the precise in situ characterization of the spatial profile of the optical intensity that modulates the electron beam. Visualizing weak-phase objects in electron microscopy relies on converting subtle phase shifts into measurable intensity variations. Techniques such as off-axis electron holography, Zernike phase contrast, in-line phase contrast and laser-based phase modulation have been developed to achieve this transformation^49^. These advanced methods are especially promising for imaging biological specimens, where enhanced phase sensitivity is crucial for revealing fine structural details without the need for staining or exposure to high electron doses. Here we introduce ultrafast four-dimensional scanning transmission electron microscopy (U4DSTEM) method, which enables the direct in situ mapping of light-induced electron phase modulation at the interaction plane with nanometre-scale resolution defined by the electron beam and allows the reconstruction of the underlying optical intensity profile.

## Results

We experimentally demonstrate the correction of the spherical aberration of an electron beam induced by the focusing system of a scanning electron microscope column equipped with a field-emission source. The aberration and its correction are shown via monitoring a highly magnified point-projection image of an optical standing wave, generated by two counter-propagating laser pulses in the perpendicular direction with respect to the electron beam, which serves as an etalon sample. The aberration is corrected via spatial phase modulation of the electron beam induced by interaction with a shaped pulsed light beam, propagating in the opposite direction to the electrons, which we refer to as the optical field electron modulator (OFEM). Specifically, we apply Laguerre–Gaussian (LG) mode of charge one. Although the LG beam acts similar to a convex lens close to the centre of the beam, it fundamentally differs from conventional electron lenses by introducing strong spherical aberration of negative sign, enabling the compensation of spherical aberration induced by the traditional electron optics.

**Fig. 1:**
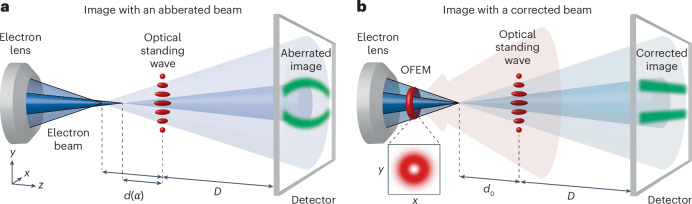
Principle of light-based corrector of spherical aberration of a focused electron beam. **a**, Focal distance in an aberrated beam is a function of the convergence angle of rays. A point-projection image of the equidistant fringes of an optical standing wave generated on the detector is distorted due to angle-dependent magnification *M*(*α*) = *D*/*d*(*α*), where *α* is the deviated angle. **b**, Application of an LG beam of charge one (its intensity distribution is shown in the inset) upstream of the focus (OFEM) leads to the correction of spherical aberration. All rays are focused to a common focal point, resulting in constant magnification across the detector and undistorted image of equidistant straight fringes. The distance *d*_0_ between the geometrical focal point and the optical standing wave does not depend on the angle anymore. The electron beam and laser fields are pulsed. The semi-transparent red cone schematically represents the converging LG laser beam that forms the OFEM. The blue cones and shaded cones represent the electron beam.

**Extended Data Fig. 1:**
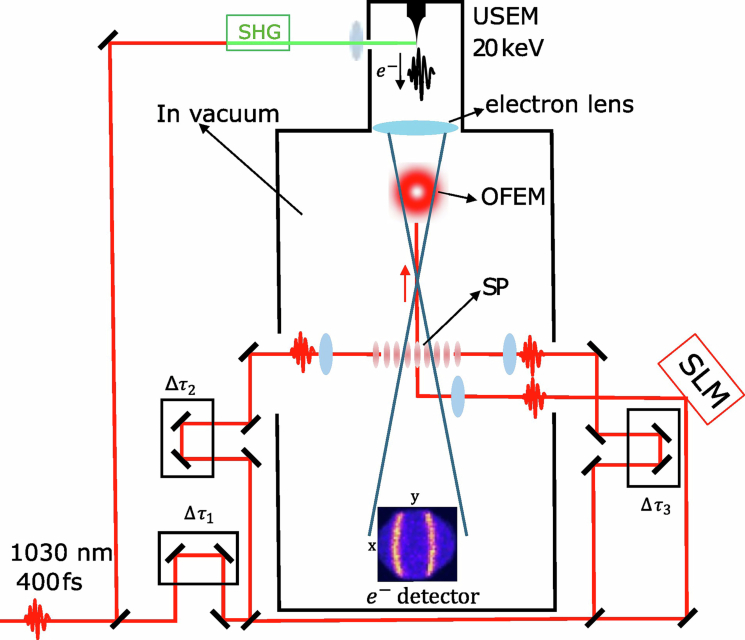
Schematic of the experimental setup for spherical aberration correction of electrons using light. 20 keV pulsed electron beam (*τ*_e_ = 680 fs) generated by linear photoemission process by the second harmonic frequency of a femtosecond laser (*λ*_L_ = 515 nm, ${\tau }_{l}$ = 400 fs) is focused by the objective lens of an electron microscope column. The aberrated electron beam interacts with a LG beam at the OFEM plane generated using a spatial light modulator (SLM). Subsequently, the electron pulses reach the sample plane (SP), where they interact with an optical standing wave, which introduces additional phase modulation. Point-projection electron images of the optical standing wave with high magnification (see inset) measured using a 2D hybrid-pixel detector are used to evaluate the spherical aberration coefficient of the electron beam, which corresponds to the curvature of the measured fringes at the detector. An effective correction at the OFEM plane results in straight fringes in the detected image, indicating that the magnification is uniform across all propagation angles. The schematic is not to scale.

Figure 1 illustrates the experimental setup of a light-based electron aberration corrector (a detailed description of individual parts of the setup can be found in Methods and Extended Data Fig. 1). Figure 1a shows the point-projection microscopy of the optical standing wave measured with an aberrated electron beam. The standing wave results from an interference of two counter-propagating pulsed Gaussian beams, which can be approximated as plane waves near the focus. This results in a pattern resembling a perfect etalon sample, with straight, parallel optical fringes spaced by half the wavelength of light used to generate the standing wave. When the electrons propagate through the optical standing wave, the rays become focused in one direction in the local minima of the ponderomotive potential and defocused in its maxima. The optical standing wave, thus, acts as an equidistant series of cylindrical lenses for electrons. As a result, when the intensity of the standing wave is appropriately chosen to focus the electrons on the detector plane, fringes appear at the downstream detector. We note that the optical standing wave can be viewed as a one-dimensional lens array which, together with the two-dimensional detector, offers an analogy to an electron optical counterpart of the Shack–Hartmann wavefront sensor used in laser optics^50^. Spherical aberration with a positive sign, which is present in all electromagnetic lenses with cylindrical symmetry^2^, causes a shift in the focal point closer to the electron lens with an increasing angle between the electron trajectory and the beam axis. This leads to angle-dependent magnification *M*(*α*) = *D*/*d*(*α*) = *D*/(*d*_0_ + *C*_s_*α*^2^), where *D* is the distance between the optical standing wave and the detector, *d*_0_ is the distance between the geometrical focal spot and the optical standing wave, and *d*(*α*) represents the angle-dependent path length between the focal spot and the standing wave, incorporating spherical aberration. The image acquired with the aberrated electron beam, thus, shows curved fringes with their curvature directly related to the value of the spherical aberration coefficient *C*_s_. Once the aberration correction by OFEM is applied (Fig. 1b), all electron rays are focused to a single focal point, leading to straight fringes at the detector.

**Fig. 2:**
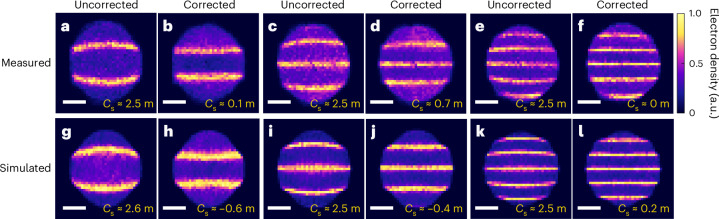
Electron point-projection images of an optical standing wave acquired with an electron beam with and without spherical aberration. Top row: experimental images measured with the aberrated electron beam (**a**, **c** and **e**) are compared with the images acquired with the electron beam corrected by OFEM (**b**, **d** and **f**). The corresponding values of the spherical aberration coefficient obtained by the procedure described in Supplementary Fig. 2 are shown in the corresponding panels. The data were obtained with three slightly different focal distances of the objective lens, leading to different magnifications. Bottom row: numerical simulations of images acquired with aberrated (**g**, **i** and **k**) and corrected (**h**, **j** and **l**) electron beams calculated with the same parameters, which were used in the experiments. Scale bars, 550 µm (**a**–**l**). All panels share the same colour scale for direct visual comparison.

In Fig. 2, we present the experimental results (Fig. 2a–f) alongside the corresponding simulations (Fig. 2g–l). The images were acquired using an aberrated electron beam (Fig. 2a,c,e,g,i,k) and an aberration-corrected beam (Fig. 2b,d,f,h,j,l). Data were collected at three different magnifications, approximately ×1,288, ×937 and ×714, achieved by varying the working distance of the microscope (distance *d*_0_).

The fringes were fitted with an analytical function to obtain the value of *C*_s_, which is *C*_s_ ≈ 2.5 ± 0.1 m for the aberrated beam and *C*_s_ ≈ 0.1 ± 0.1 m for the corrected beam at the highest magnification. The uncertainty in *C*_s_ is estimated based on the standard error of the fitted fringe positions, calculated from the residuals of the fit and the sensitivity of the model to changes in *C*_s_ (Supplementary Equation (3)). We note that for each image, the magnification, achieved by shifting the focal spot of the electron beam, also changes the beam size at the interaction plane with the OFEM. As a result, the defocus of the laser beam and its temporal overlap with the electron pulse must be carefully readjusted. The residual *C*_s_ observed in the experimental data (Fig. 2d) probably reflects imperfect laser alignment under these conditions. The results confirm that the OFEM with the LG beam effectively compensates the spherical aberration of the electron beam.

One of the crucial requirements for the successful implementation of light-based aberration correctors in electron microscopy is a precise characterization of the spatial distribution of phase modulation induced to the electrons. To obtain this information directly in situ, we turned off the optical standing wave, focused the electron beam to the OFEM plane and used U4DSTEM imaging^51^. We map the transverse momentum transfer imparted by the OFEM by scanning the electron focal spot and measure the centre of mass of electron distribution on the detector. Because the transverse momentum transfer is directly proportional to the gradient of the optical intensity integrated along the electron trajectory, such a measurement represents a direct spatial mapping of the strength of interaction between electrons and light.

**Fig. 3:**
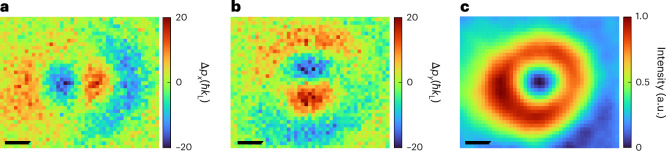
Spatial distribution of the electron phase modulation induced by the light-based aberration corrector. **a**,**b**, Measured *x* (**a**) and *y* (**b**) components of the transverse momentum change in the electrons as a function of the electron-beam position in the OFEM plane, as obtained using the U4DSTEM technique. **c**, Reconstructed profile of the ponderomotive potential integrated along the electron trajectory of the LG mode obtained from the data in **a** and **b**. Scale bars, 2 µm (**a**–**c**).

From two-dimensional gradient maps of the *x* and *y* components of the transverse momentum change in the electrons (Fig. 3a,b), we reconstructed the spatial profile of the phase modulation imprinted to the electron beam by OFEM (Fig. 3c). The reconstruction was performed by applying a Fourier-based Poisson solver to the measured beam-shift gradients^52^ (Supplementary Equation (11)). The waist of the LG beam, *w*_LG_ = 4.45 µm, was extracted by fitting the reconstructed intensity distribution (Fig. 3c) and subsequently used in the calculation of the ponderomotive phase shift in equation (2) for all the simulated images. These results demonstrate the ability of U4DSTEM to resolve light-induced momentum distributions and reconstruct optical field profiles with spatial resolution, which is not limited by light diffraction as in the case of optical imaging systems^38^ but only by the resolution of the electron microscope itself. We note that the resolution in Fig. 3c is limited by the pixel size of 300 nm, but it can go down to ~20 nm (ref. ^51^). The deviation from an ideal LG beam observed in Fig. 3c arises from aberrations induced in the optical setup (mirror with a hole, focusing lens). However, the inner part of the intensity distribution with a radius of <3 µm that interacts with the electrons remains almost undistorted.

**Fig. 4:**
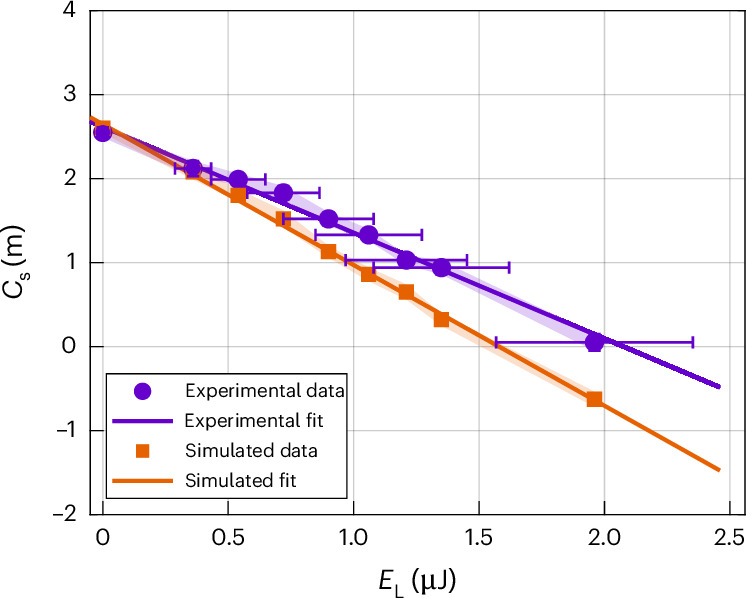
Electron spherical aberration coefficient as a function of OFEM pulse energy. *C*_s_ coefficients determined from the experimental images (violet circles) and simulated images (orange squares) as a function of OFEM pulse energy. Data are presented as individual values ± standard error (vertical shadow regions) of the fit used to extract *C*_s_ calculated from the residuals and sensitivity of the cubic fringe fitting model (Supplementary Equation (3)). The horizontal error bars indicate the estimated experimental uncertainty in the laser pulse energy delivered to the OFEM plane.

To quantify the OFEM tunability, we varied the pulse energy *E*_L_ used for compensation. In Fig. 4, we show the extracted *C*_s_ values from the experimental and simulated images with the highest magnification. The full *C*_s_ correction is reached for *E*_L_ ≈ 2 μJ.

Details on the *C*_s_ extraction procedure and the experimental images used to obtain the data shown in Fig. 4 are provided in Supplementary Figs. 1 and 2. The residual *C*_s_ observed in the simulations after correction is primarily attributed to an estimated 20% in the laser pulse energy delivered to the OFEM plane, which also accounts for the difference in slopes between the theory and experiment (Fig. 4), as both effects scale with the same factor. Furthermore, at lower image magnifications, the reduced pixel resolution limits the precision of curvature fitting in point-projection electron images, contributing to the observed undercompensation.

## Numerical modelling

To quantitatively interpret the experimental results, we use a ray optics model that captures the propagation of the aberrated electron beam and its interaction with both OFEM and optical standing wave. In this approach, the electron beam is treated as an ensemble of classical rays, and wave interference or coherence effects are neglected. The ray-tracing algorithm is initialized at the OFEM plane, with the initial propagation angles of the electrons *θ*_0_(*x*) = –*x*/*d*_c_ and *θ*_0_(*y*) = –*y*/*d*_c_, where *d*_c_ is the distance from the OFEM plane to the geometrical focus of the electron beam, and *x* and *y* are the transverse spatial coordinates within the circular beam profile. The propagation of rays between the interaction planes and the detector is modelled using the free-space propagation matrix formalism. The local change in propagation direction is calculated from the phase gradient using the relation δ*θ*_*i*_ = 1/*k*_e_∇*φ*_*i*_, where *i* denotes the contribution from spherical aberrations, the OFEM or the optical standing wave. Here *k*_e_ = 2π/*λ*_e_ is the electron wavenumber, with *λ*_e _*=* *h/*(*γm*_e_*ν*) denoting the relativistic electron wavelength. In this expression, *ν* is the electron velocity, *m*_e_ is the electron mass and $\gamma =1/\sqrt{1-{v}^{2}/{c}^{2}}$ is the Lorentz factor accounting for relativistic effects.

The wavefront error of the electron beam due to the spherical aberration is defined as^1^

1 $${{{\varphi }}}_{{\rm{a}}}\left({\rm{\theta}}\left(x,y\right)\right)=-\frac{{{\uppi}}}{2{{{\lambda}}}_{{\rm{e}}}}{C}_{{\rm{s}}}{{{\theta }}}^{4}\left(x,y\right),$$

where *θ*(*x*, *y*) is the semi-convergence angle.

The analytical expression for the phase shift acquired by an electron interacting with a counter-propagating LG laser focal spot is given by^37^^,38^

2 $${{{\varphi }}}_{{\rm{l}}}\left(x,y\right)\approx - {\frac{{{\alpha}}}{2{{\uppi }}\left(1+{{\beta}}\right)}\frac{{E}_{{\rm{L}}}{\lambda }_{{\rm{L}}}^{2}}{{E}_{{\rm{e}}}}\frac{{g}_{\text{LG}}^{2}\left(x,y\right)}{{\int }_{-\infty }^{\infty }{\int }_{-\infty }^{\infty }\text{d}x\text{d}y\;{g}_{\text{LG}}^{2}\left(x,y\right)}}.$$

The parameters in the expression are defined as follows: *α* is the fine-structure constant, *β* = *v*/*c* represents the electron velocity normalized to the speed of light and *λ*_L_ denotes the laser wavelength. The relativistic energy of the electron is given by *E*_e_ = *γm*_e_*c*^2^. Furthermore, the spatial distribution of the laser intensity is given by ${g}_{\text{LG}}^{2}\left(x,y\right)=(\;{\rho }^{2}/{w}_{{\rm{LG}}}^{2})\exp (-2{\rho^{2}} /{w}_{{\rm{LG}}}^{2})$, where $\rho =\sqrt{{x}^{2}+{y}^{2}}$ denotes the radial coordinate.

When electrons propagate through the optical standing wave formed by two counter-propagating Gaussian laser pulses propagating in *x* direction, they acquire a phase shift that can be calculated as^29^

3 $$\begin{array}{l}{{{\varphi }}}_{{\rm{g}}}\left({x}^{{\prime} },{y}^{{\prime} }=0,{z}^{{\prime} }=0,t\right)=\displaystyle\frac{{e}^{2}{\lambda }_{{\rm{L}}}^{2}{I}_{0}}{16{\uppi }^{2}{m}_{{\rm{e}}}{\epsilon }_{0}{c}^{3}{{\hslash }}}\\ {\int }_{-\infty }^{\infty }\left[\exp \left(-\displaystyle\frac{{\left(t-\displaystyle\frac{{x}^{{\prime} }}{c}\right)}^{2}}{2{w}_{{\rm{t}}}^{2}}\right)+\exp \left(-\displaystyle\frac{{\left(t+\displaystyle\frac{{x}^{{\prime} }}{c}\right)}^{2}}{2{w}_{{\rm{t}}}^{2}}\right)\right.\\+\;\left.2\exp \left(-\displaystyle\frac{{t}^{2}}{2{w}_{{\rm{t}}}^{2}}\right)\exp \left(-\displaystyle\frac{{x}^{{\prime} 2}}{2{w}_{{\rm{t}}}^{2}{c}^{2}}\right)\cos \left(2{k}_{{\rm{L}}}{x}^{{\prime} }\right)\right]{{\rm{d}}t}\end{array}.$$

Here *I*_0_ denotes the peak intensity of each individual laser pulse; ${k}_{{\rm{L}}}=\frac{2\uppi }{{\lambda }_{{\rm{L}}}}$ is the laser wavenumber; the pulse duration is given by ${\tau }_{{\rm{l}}}=2\sqrt{2\mathrm{ln}[2]}{w}_{{\rm{t}}}$; and *x*′, *y*′ and *z*′ are the spatial coordinates in the plane of the optical standing wave, which we refer to as the sample plane (SP). The first two terms in the integral correspond to the individual counter-propagating pulses, giving only a spatially constant phase offset, whereas the third term represents the resulting optical standing wave.

## Discussion

In this work, we demonstrate the optical correction of third-order spherical aberration in a conventional electron lens using an LG beam with charge one. This method can be extended to correct not only the third-order spherical aberration but also higher-order electron aberration by using a gradient descent optimization algorithm to iteratively refine the laser wavefront using an SLM capable of both phase and amplitude modulation. The optimization process can be guided by the acquired electron image itself, enabling the real-time auto-tuning of the optical field to maximize image quality^53^.

The efficiency of spherical aberration correction depends strongly on the spatial extent of the shaping region. Specifically, the laser pulse energy required to compensate a certain value of *C*_s_ scales quadratically with the electron-beam radius in the interaction plane *w*_e_, which also determines the radius of the laser beam used for OFEM, *w*_LG_ (the ratio *w*_e_/*w*_LG_ ≈ 0.67 should be maintained for compensation of the third-order spherical aberration without introducing higher-order aberration terms). This is a consequence of the linear dependence of the induced ponderomotive phase shift on the local intensity of light.

The transverse coherence of the electron beam is not altered by the interaction because it is coherent and elastic. For integration into a transmission electron microscope, the OFEM can be positioned upstream of the sample, as experimentally demonstrated in previous studies that achieved pre-sample phase modulation using optical fields near thin films^25,26^, enabling wavefront shaping without directly perturbing the specimen.

Standard high-resolution scanning electron microscopes achieve a best spatial resolution of about 0.5 nm, which is insufficient to resolve individual atoms in solid-state samples. Assuming a maximum electron energy of 30 keV, the corresponding de Broglie wavelength is approximately 7 pm. With an electron-beam divergence angle of 50 mrad, the theoretical resolution limit is about 70 pm, which has previously only been achieved using a multipole aberration corrector^54^. As ultrafast electron microscopes^55,56^ combine atomic spatial resolution with femtosecond temporal precision to visualize dynamic processes at the space-time scale, they could greatly benefit from this light-based aberration correction approach, which offers a compact and tunable alternative to traditional multipole correctors.

The crucial points for applicability of the light-based electron optical correctors in standard electron microscopes are the spatial stability of the corrector and the possibility to work in the continuous regime. The spatial stability of OFEM is determined by the combination of the pointing stability of the optical beam and the mechanical stability of the focusing lens and other optical components. For reaching sub-nanometre spatial resolution, the OFEM also has to be spatially stabilized with the same precision, which represents a major challenge for future research. Continuous operation requires enhancing the electron–light interaction strength, which can be achieved using a resonant optical cavity to accumulate sufficient optical intensity^32^. Although cavity powers on the order of 100 kW have been previously demonstrated, the power required to correct spherical aberrations in transmission electron microscopes could be reduced by aligning the cavity coaxially with the electron beam. In the current configuration, the effective interaction distance *d*_int_ ≈ 26 μm is limited by the pulsed nature of electrons and light^38^. By contrast, a continuous-wave operation would enable an extended interaction distance. Another parameter influencing the OFEM efficiency via the amplitude of the phase shift induced by the ponderomotive interaction is the wavelength of light used in OFEM. Longer wavelengths enhance the interaction strength for a fixed beam waist, although maintaining a constant beam radius with increasing wavelength requires an increase in the numerical aperture of the focusing setup, which may pose practical challenges. We note that the selection of wavelength and numerical aperture also influences the spatial resolution of the OFEM, which is ultimately limited by light diffraction.

In summary, we have demonstrated that the dominant electron lens aberration—specifically the third-order spherical aberration—can be corrected via free-space interaction with a shaped light beam.

In addition, we introduced the U4DSTEM technique that allows the in situ characterization of the phase profile imprinted on the electrons, which is essential for the further development of any structured light-based electron phase modulators. Furthermore, the optical standing wave enables the in situ calibration and assessment of electron optical distortions using a known optical phase etalon, providing a powerful tool for precise wavefront metrology within these setups.

Unlike conventional aberration correctors requiring multiple lenses, which also offer real-time feedback-based aberration compensation, our single-plane light-based approach may offer a pathway towards miniaturized aberration correction systems. With continued progress in adaptive optics and artificial-intelligence-driven feedback control, dynamically tunable, light-based optical elements may become integral to next-generation electron microscopy systems, enabling real-time, reconfigurable beam shaping with unprecedented precision and flexibility.

## Methods

In our experimental setup (Extended Data Fig. 1), we generate ultrashort electron pulses via laser-triggered photoemission from a Schottky field emission gun integrated into a Verios 5 UC scanning electron microscope (Thermo Fisher Scientific). We used the second harmonic (515 nm) of a femtosecond (400 fs, 50 kHz) laser pulse to trigger photoemission from a Schottky field emitter, producing single-electron pulses that reach the SP with a duration of approximately 680 fs at an energy of 20 keV with a maximum semi-convergence angle of *α*_max_ = 5 mrad (ref. ^57^). The transverse spatial properties of the pulsed electron beam do not change significantly compared with the continuous emission mode.

As electrons propagate towards the SP, they accumulate spherical aberration, with the probe-forming lens located approximately 5 cm upstream of the SP. Due to the absence of an aperture, the electron-beam radius at the lens is estimated to be 225 µm, which contributes to the strength of the third-order spherical aberration. The resulting wavefront distortion is characterized by a spherical aberration coefficient of *C*_s_ ≈ 2.5 m. To compensate for aberrations, the electron wavefront is phase modulated at the OFEM plane through interaction with an LG beam, generated by an SLM (HOLOEYE Photonics, PLUTO 2.1). The converging electron beam, with a radius of approximately 3 µm, illuminates only the central region of the LG mode. After this initial interaction, the electrons pass through a crossover and propagate to the SP, where they interact with a second optical field in the form of a standing wave, which is used as an etalon sample. The standing wave is generated by focusing two counter-propagating pulsed laser beams with a wavelength of 1,030 nm and pulse duration of 400 fs. The beam radius in the focus is *w*_0_ = 10 µm. The resulting electron image of the standing wave is recorded on a position-sensitive detector (Timepix 3, AdvaScope) located 17 cm downstream of the SP. The distance between the OFEM and SP is approximately 0.74 mm for the data shown in Fig. 2, and all the experimental point-projection images were acquired with an exposure time of 5 s.

The standing wave serves as an etalon sample with an exact spacing of its interference maxima and perfectly flat phase front (we only observe a small part of the interference pattern of the optical standing wave with the size of ~700 nm in the data shown in Fig. 1), enabling imaging near the electron crossover at high magnifications, where spherical aberrations are the most pronounced, without interfering with the OFEM. The *λ*_L_/2 periodicity of the standing wave creates high spatial phase gradients, resulting in pronounced fringes at the detector plane using *E*_L_ < 1 µJ per counter-propagating laser pulse and a numerical aperture of NA = 0.08.

The OFEM is brought on axis with the electron beam using a pellicle beamsplitter (BP145B3, Thorlabs; not shown in Extended Data Fig. 1) with a hole in the centre. This hole allows the electron beam to pass through unobstructed, enabling the co-linear alignment of the OFEM. The hole is fabricated in-house by diffracting the laser focus (NA = 0.13) into a circular pattern with a radius of 150 µm using an SLM and applying pulses with an energy of 6 µJ. The NA of the OFEM is 0.13.

The timing between the OFEM and the electron pulse is adjusted using the first delay stage. The second delay stage controls the relative timing of the counter-propagating laser pulses that form the standing wave, whereas both second and third delay stages are used to synchronize the standing wave with the arrival of the electron pulse at the SP.

## Online content

Any methods, additional references, Nature Portfolio reporting summaries, source data, extended data, supplementary information, acknowledgements, peer review information; details of author contributions and competing interests; and statements of data and code availability are available at 10.1038/s41566-025-01760-8.

## Supplementary information

Supplementary Information Supplementary Figs. 1 and 2, Equations (1)–(11) and Text.

## Data Availability

The data that support the findings of this paper are available via Zenodo at 10.5281/zenodo.16532408 (ref. ^58^).

## Code availability

All computational codes used to generate the data presented in this study are available from the corresponding authors upon reasonable request.
