# Supplementary material for: Light-based electron aberration corrector
Source: Nat Photonics. 2025 Sep 23;19(12):1309–14. doi: 10.1038/s41566-025-01760-8 (PMC12672372; doi:10.1038/s41566-025-01760-8)
Supplement: Supplementary file 1 — Supplementary Figs. 1 and 2, Equations (1)–(11) and Text. [file 41566_2025_1760_MOESM1_ESM.pdf]

nature photonics

Supplementary information https://doi.org/10.1038/s41566-025-01760-8

Light-based electron aberration corrector

In the format provided by the authors and unedited

Supplementary Text

1. Calculating the spherical aberration coefficient of the electron beam

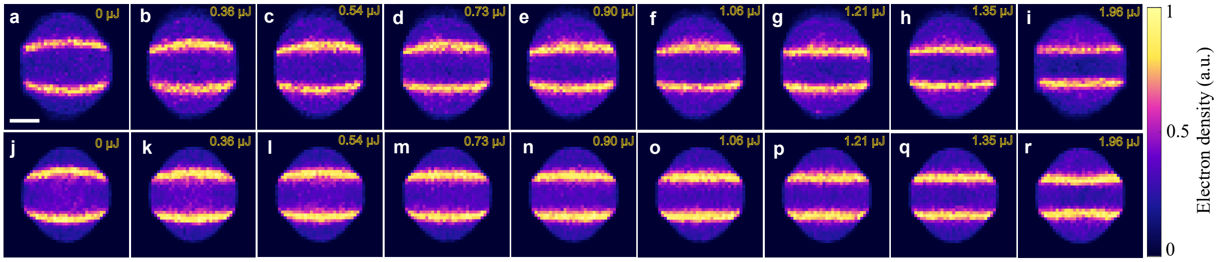

Supplementary Figure S1|Measurement of spherical aberration correction as a function of OFEM pulse energy. Panels (a–i) show experimental electron point-projection images of an optical standing wave acquired at increasing OFEM pulse energies. Panels (j–r) show the corresponding simulations. As the pulse energy increases, the curvature of the fringes progressively straightens, indicating effective aberration compensation. All images of the standing wave are shown in their raw form, without any image processing. Panels a-r share the same scale as panel a, where a scale bar of 550 μm is shown.

Figure S1 presents a direct comparison between the experimental point-projection electron images (panels a–i) and the corresponding simulations (panels j–r) across a range of pulse energies. At low energies, the fringes appear notably curved due to the uncorrected spherical aberration. As the OFEM pulse energy increases, the curvature of these fringes progressively straightens, indicating that the aberration is being compensated. At a specific pulse energy, the fringes become nearly flat (panels I/R), marking the point of optimal aberration compensation.

The pulse energies in the focal spot of the full LG beam corresponding to images a–i and j–r are, in units of μJ: 0, 0.36, 0.54, 0.72, 0.90, 1.06, 1.21, 1.35, and 1.96. It should be noted that the electron beam interacts with only a fraction of the total pulse energy, as it is confined to the central region of the LG beam.

The C_s was determined using the following procedure: The images were converted to grayscale and smoothed column-wise to enhance peak detection. Subpixel stripe positions were determined by fitting a 1D Gaussian to a 5-pixel window centered around the brightest peaks in each image column, enabling accurate localization of the top and bottom electron fringes. The transverse fringe positions

were converted from pixel to metric units using the known pixel size of the detector. Quadratic functions were fitted to the top and bottom fringe positions as a function of horizontal coordinate, with the beam center, identified as the location of maximum fringe separation, establishing the origin of the coordinate system used in subsequent analysis.

After centering, a second round of cubic fits (Eq. S7) was performed to extract the curvature of the top and bottom fringes relative to the beam center; for each image, C_s was computed separately from both fringes and their mean value was recorded (see Fig. S2). This analysis was performed on experimental images a to i and simulated images j to r from Fig. S1, and the resulting C_s values are plotted in Fig. 4 in the main manuscript. To estimate the uncertainty of the C_s, we fit the cubic function (Eq. S7) independently to the top and bottom electron fringe positions. For each fit, the residuals are used to compute the mean squared error (MSE). The sensitivity of the model to C_s is evaluated numerically using finite differences. The standard error is then calculated as:

$$\sigma_{C_\mathrm{s}} = \sqrt{\frac{\mathrm{MSE}}{\sum_i \left(\frac{dy_i}{dC_\mathrm{s}}\right)^2}}, \tag{S1}$$

where y_i (equivalent to y_det used later) is the model-predicted stripe position for the i-th data point, and $\frac{dy_i}{dC_\mathrm{s}}$ is its numerical derivative with respect to C_s, computed via:

$$\frac{dy_i}{dC_\mathrm{s}} \approx \frac{f(C_\mathrm{s}+\delta, x_i) - f(C_\mathrm{s}, x_i)}{\delta}. \tag{S2}$$

Here, $f(C_\mathrm{s}, x_i)$ is the cubic model evaluated at position x_i, and δ is a small perturbation.

The final uncertainty is obtained by combining the top and bottom errors via root-mean-square averaging:

$$\sigma_\mathrm{final} = \sqrt{\frac{\sigma^2_{C_\mathrm{s},\mathrm{top}} + \sigma^2_{C_\mathrm{s},\mathrm{bottom}}}{2}}. \tag{S3}$$

The cubic fit function (Eq. S7) is derived based on the following considerations. The radial distance from the center of the electron beam on the detector is defined as:

$$r_\mathrm{det} = \sqrt{x^2_\mathrm{det} + y^2_\mathrm{det}}, \tag{S4}$$

where x_det and y_det are detector-plane coordinates of the fringes with respect to the center. Under the small-angle approximation, the angular deviation from the electron beam axis is $\alpha = r_\mathrm{det}/D$ with D denoting the distance from the optical standing wave to the detector.

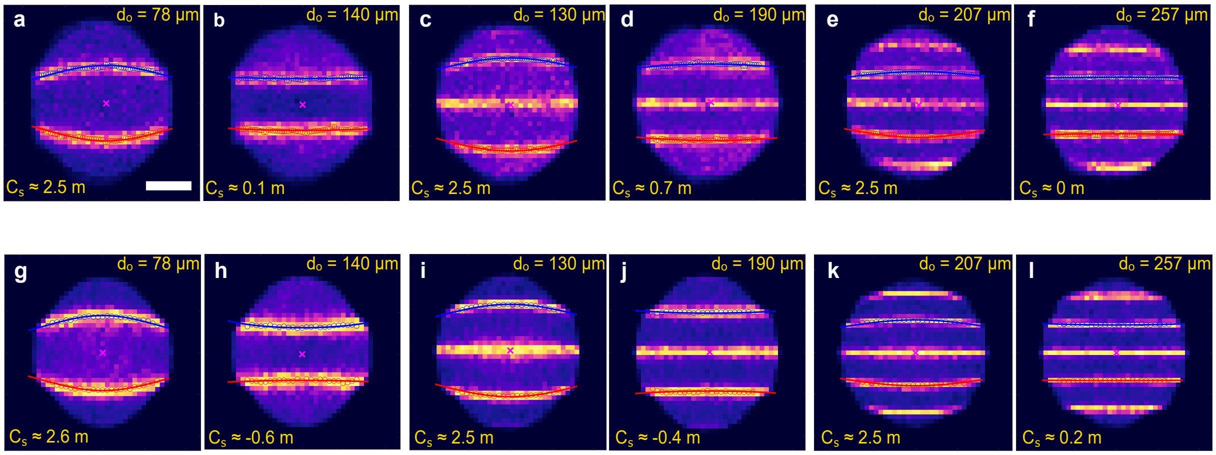

Supplementary Figure S2|Electron fringe fit curvature before and after OFEM correction, shown for experimentally acquired images at different magnifications. Panels a and b show the experimental fringe patterns with the OFEM off and on, respectively, while Panels c and d present their corresponding simulations. In each case, the positions of the top and bottom fringes were fitted with cubic polynomials (Eq. S7) to quantify the curvature and extract the C_s value. The slight asymmetry between the top and bottom curves in each image can be attributed to a small lateral displacement of the standing wave relative to the center of the electron beam. Note that for panels c to f, and i to l, the denominator of Eq. S6 changes from $\lambda_L/2$ to λ_L. Panels a-l share the same scale as panel a, where a scale bar of 550 μm is shown.

The longitudinal shift of the focal distance due to spherical aberration can be expressed as:

$$\Delta z(\alpha) = C_s\alpha^2. \tag{S5}$$

With d_0 as the distance from the standing wave to the geometrical electron cross over (focal point for on-axis rays), the magnification of two fringes displaced from the center becomes:

$$M(\alpha) = \frac{D + d_0 + C_s\alpha^2}{d_0 + C_s\alpha^2} \doteq \frac{D}{d_0 + C_s\alpha^2} = \frac{2y_{\text{det}}}{(\lambda_L/2)}. \tag{S6}$$

Here we used the fact that the distance between the geometrical cross over and the optical standing wave d_0 is much smaller than the distance between the optical standing wave and the detector D. The right hand side of Eq. S6 corresponds to the experimentally measured magnification obtained by dividing the measured fringe separation $2y_{\text{det}}$ by the known period of the optical standing wave $(\lambda_L/2)$. The fit function used to evaluate the curvature of the fringes and retrieve the

C_s coefficient is obtained by substituting Eqs. S4 and S5 into Eq. S6. The resulting equation has the form:

$$2C_s y_{det}^3 + \left(2d_0 D^2 + 2C_s x_{det}^2\right) y_{det} - \frac{\lambda_L}{2} D^3 = 0. \tag{S7}$$

The solution of cubic Eq. S7 is used to fit the experimentally measured positions of fringes in the detector plane. The electron defocus component associated with the LG beam results in a shift of the geometrical focal spot by only 60 μm upstream towards the objective lens.

Furthermore, we did not observe a measurable contribution from fifth-order spherical aberration. This is because the electron beam interacts with the central region of the LG beam, where the influence of higher-order terms in the Taylor expansion of the ponderomotive potential is minimal. Consequently, any fifth-order contributions were below our detection sensitivity within the experimental conditions used.

2. OFEM Spatial Intensity Distribution Reconstruction from the Measured Gradients

Reconstructing a scalar field such as the intensity $I(x, y)$ from its spatial gradients is a classical inverse problem in imaging physics. Let $I(x, y)$ denote a twice-differentiable scalar field whose gradient is known:

$$\nabla I(x, y) = \left(\frac{\partial I(x, y)}{\partial x}, \frac{\partial I(x, y)}{\partial y}\right). \tag{S8}$$

To recover the original intensity, one computes the divergence of this gradient field, which yields the Laplacian of $I(x, y)$:

$$\nabla^2 I(x, y) = \nabla \cdot (\nabla I(x, y)). \tag{S9}$$

Applying the two-dimensional Fourier transform to both sides of the equation and using the identity that the Fourier transform of the Laplacian is equivalent to multiplication by the spatial frequency variables in the x and y directions, $-(k_x^2 + k_y^2)$, we obtain

$$-(k_x^2 + k_y^2)\, \mathcal{F}\{I(x, y)\} = \mathcal{F}\{\nabla \cdot (\nabla I(x, y))\}. \tag{S10}$$

Finally, taking the inverse Fourier transform provides the reconstructed intensity distribution in real space:

$$I(x, y) = \mathcal{F}^{-1}\left\{\frac{-\mathcal{F}\left[\nabla \cdot (\nabla I(x, y))\right]}{k_x^2 + k_y^2}\right\}. \tag{S11}$$

To avoid division by zero at the origin of Fourier space, where $k_x = k_y = 0$, the corresponding component of the Fourier transform is set to zero manually. This removes the undefined global offset, which cannot be recovered from gradient data alone.

The U4DSTEM experiment was conducted using a relatively long objective lens working distance of 15.5 mm, with a 64 μm diameter aperture and the microscope operating at its highest current setting. Under these conditions, the electron beam radius at focus is limited by aberrations in the electron optics to about 20 nm, which defines the intrinsic spatial resolution. However, in the measurements presented in Fig. 3 in the main manuscript, the effective resolution was constrained by the scan step size of the electron focal spot, which was about 300 nm. The SLM was used to correct for deviations from the ideal LG intensity distribution. The long acquisition time required for the data in Fig. 3C (30 minutes) made it impractical for use in an active feedback loop. Nevertheless, since the electron beam interacts only with the central region of the LG beam (radius $< 3\,\mu$m), the profile in this area can be approximated as aberration-free.
